# Towards Recycling of LLZO Solid Electrolyte Exemplarily Performed on LFP/LLZO/LTO Cells**

**DOI:** 10.1002/open.202100274

**Published:** 2022-02-23

**Authors:** Mohammad Ali Nowroozi, Aamir Iqbal Waidha, Martine Jacob, Peter A. van Aken, Felicitas Predel, Wolfgang Ensinger, Oliver Clemens

**Affiliations:** ^1^ Technical University of Darmstadt Institut für Materialwissenschaft Alarich-weiss-Straße 2 64287 Darmstadt Germany; ^2^ University of Stuttgart Institut für Materialwissenschaft Heisenbergstraße 1 70569 Stuttgart Germany; ^3^ Max Planck Institute for Solid State Research Stuttgart Centre for Electron Microscopy Heisenbergstraße 1 70569 Stuttgart Germany

**Keywords:** all-solid-state lithium-ion batteries, circular economy, hydrometallurgy, Li_7_La_3_Zr_2_O_12_, recycling

## Abstract

All‐solid‐state lithium ion batteries (ASS‐LIBs) are promising due to their safety and higher energy density as compared to that of conventional LIBs. Over the next few decades, tremendous amounts of spent ASS‐LIBs will reach the end of their cycle life and would require recycling in order to address the waste management issue along with reduced exploitation of rare elements. So far, only very limited studies have been conducted on recycling of ASS‐LIBS. Herein, we investigate the recycling of the Li_7_La_3_Zr_2_O_12_ (LLZO) solid‐state electrolyte in a LiFePO_4_/LLZO/Li_4_Ti_5_O_12_ system using a hydrometallurgical approach. Our results show that different concentration of the leaching solutions can significantly influence the final product of the recycling process. However, it was possible to recover relatively pure La_2_O_3_ and ZrO_2_ to re‐synthesize the cubic LLZO phase, whose high purity was confirmed by XRD measurements.

## Graphical abstract

**Recycling of solid‐state batteries**, which are expected to become extremely important in the energy industry over the next decade, is often very complex due to the solid electrolyte. Here, recycling of all‐solid‐state lithium ion batteries has been approached by a combination of mechanical/hydrometallurgical methods. Promising results confirm the recovering of the solid‐electrolyte.

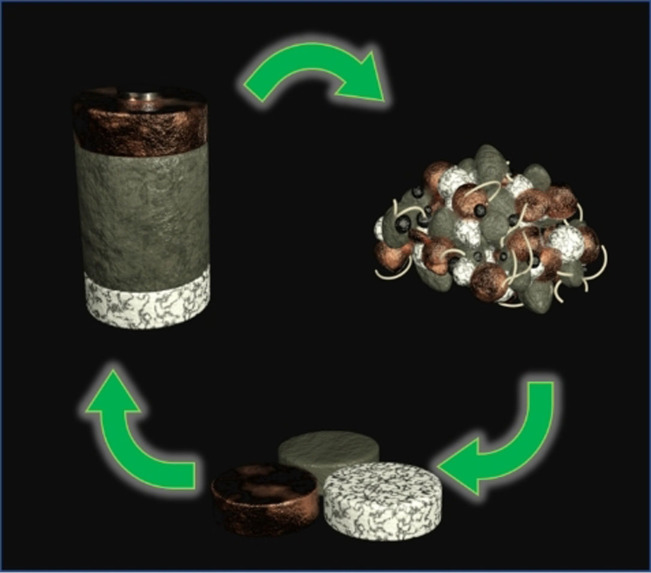

## Introduction

The consumption of lithium ion batteries (LIBs) has significantly increased mainly due to incredibly fast‐growing portable electronics and e‐mobility industry. In this respect, we are on track to produce millions of tons of spent LIBs that are on the verge of their cycle life. By recycling the spent LIBs, we can manage the battery waste as well as preventing/reducing damages to the environment due to extraction of precious elements used to manufacture LIBs. Obviously, a battery has a perfect elemental composition to refabricate the same battery again. As yet, less than 5 % of LIBs are recycled,^[1]^ whereas, for example, lead‐acid batteries are almost fully recycled.^[2]^ Fortunately, several research studies have recently been performed on recycling of conventional LIBs (with liquid electrolytes) at laboratory scale with a main focus on recycling of the metal elements of the current collectors (Cu and Al) as well as the cathode materials such as LiCoO_2_ (LCO),^[3]^ LiNi_0.8_Mn_0.1_Co_0.1_O_2_ (NMC),^[4]^ and LiFePO_4_ (LFP).^[5]^

It is expected that in the near future, all‐solid‐state lithium ion batteries (ASS‐LIBs) will enter the market of the energy industry since they offer higher safety and energy density^[6]^ as compared to conventional LIBs. However, most of the studies about recycling of LIBs are devoted to conventional liquid‐based electrolyte LIBs, while little attention has been paid to recycling of new emerging ASS‐LIBs.^[7]^ Among these, the recycling of sulfide‐based ASS‐LIBs (e. g., Li|Li_6_PS_5_Cl|LiCoO_2_)^[8]^ and oxide‐based ASS‐LIBs such as LLZO (Li_7_La_3_Zr_2_O_12_)^[9]^ have been reported.

Oxide‐type Li‐ion conductors have extensively been studied in the context of solid‐electrolytes for LIBs due to their safety (non‐flammable and non‐toxic) and stability (from chemical, thermal and electrochemical aspects).[^6b^, ^10^] Among them, garnet‐type LLZO seems to be one of the most promising solid‐state electrolyte for ASS‐LIBs,^[11]^ since it is chemically stable towards metallic Li and hence allows for the direct use of Li as a negative electrode and also provides a very high ionic conductivity in the order of 10^−3^ S cm^−1^ at room temperature.^[12]^ Therefore, it is plausible that, in the future, LLZO might become a widely used solid Li‐ion conductor to make solid electrolytes, a kind of solid separator (as a thin layer) or a filler within a polyethylene oxide (PEO) matrix (composite electrolytes)^[13]^ to improve the chemical stability of other solid‐state electrolytes. In this respect, investigations on recycling of LLZO in the context of ASS‐LIBs are necessary. In case of recycling of LLZO used as the filler within composite electrolytes, the organic PEO component can be removed by either dispersing the mixture in a heavy liquid like 1,1,2,2‐tetrabromoethane and allowing for the physical separation of LLZO from the organic PEO due to its higher density or by the thermal treatment of the composite electrolytes to remove the organic PEO component (PEO decomposes at temperatures above 320–330 °C),^[14]^ which has previously been followed by removing the organic binder (polyvinylidene fluoride (PVDF)) used in cathode composites.^[15]^ After this initial step, the recycling of LLZO can be carried out further.

Theoretically, recycling of LLZO by a hydrometallurgical approach appears simple: the garnet‐type LLZO is somehow dissolved, then different elements will be extracted upon alkali precipitation at different pH levels, and finally Li could be recovered by a substitution reaction (e. g., using Na_2_CO_3_ to substitute Na and Li and form Li_2_CO_3_) at sufficiently high pH levels.^[9]^ However, from a practical point of view, it is not as simple as it seems: First of all, apart from the solid electrolyte, a full cell of an ASS‐LIB contains cathode material, anode material, binder, an electron conductor such as graphite and current collectors. This makes the system highly complex. Moreover, in most cases, the precipitation in alkaline media results in co‐precipitation of different metal elements and further separation steps are required that may significantly increase the complexity of the recycling process. Recently, research has focused on the use of organic acids for the recycling. However, such acids are suited for recycling of electrode components,^[16]^ whereas strong inorganic acids are needed for leaching and dissolution of stable electrolyte like LLZO. Finally, the products may not be obtained in useful forms and further treatments (e. g., calcination) are required to convert them into forms that can be further used to re‐produce the desired compounds. Therefore, in a hydrometallurgical recycling process, it is important to select a suitable leaching/precipitation environment to make the recycling process as economically efficient as possible.

In this work, LLZO is recycled by hydrometallurgical methods within a cell consisting of LiFePO_4_ (LFP) and Li_4_Ti_5_O_12_ (LTO) as cathode and anode materials, respectively. The main focus of the study is to recycle the LLZO solid electrolyte within such a complex system by recovering La and Zr, preferably in form of pure La_2_O_3_ and ZrO_2_. Therefore, several leaching/precipitation scenarios have been investigated for the recycling processes. This study should help to improve our understanding on the dissolution/precipitation behavior of the different components of the system, which can also be adapted to other similar cases.

## Results and Discussion

### Acid Leaching – Alkali Precipitation

To understand the effect of the concentration of the acidic solution, the (selective) leaching processes of the LFP/LLZO/LTO mixture have been performed at three different conditions according to the concentration of the HCl solution (from very high to low concentrations). A summary of the leaching/precipitation processes and the obtained products in each case will be discussed in the following. It should be taken into consideration that the electrochemical cells were made without any housing, casing and current collectors to reduce the complexity of the system. However, in real‐case scenarios, the issue of impurities arising from battery casing and current collectors is a challenge. In this respect, two strategies are usually followed for the recycling of conventional LIBs with liquid electrolyte.^[17]^ The first one would be using a pyrometallurgical approach without any pretreatment, which results in evaporation/decomposition of certain parts (e. g., plastic parts, binder, liquid electrolyte, etc.) and formation of a molten mixture of different metals and a slag.^[18]^ The second strategy would be dismantling of the housing, plastic cables, current collectors and so on, and then using hydrometallurgical methods to recover the metals.[^17^, ^19^] Clearly, for all‐solid‐state batteries, there is a lack of well‐stablished industrial state‐of‐the‐art of the casing, housing, current collectors and so on, making it a challenge to be investigated in detail in current recycling studies. However, the authors think that the case elaborated in this article can be considered as a first approximation to the scenario of a dismantled cell. In addition, the authors assume that trace impurity metals from the use of Cu and Al as current collectors (which would be the most likely impurities transferred within a dismantling process due to direct contact to the electrodes) might behave similar to Fe during the treatment processes used. To avoid any confusion, an ID number is assigned to each product after the leaching/precipitation processes (see Table S1, Supporting Information, for details).

### Leaching Using High Concentration HCl with pH<0.2

**Figure 1:**
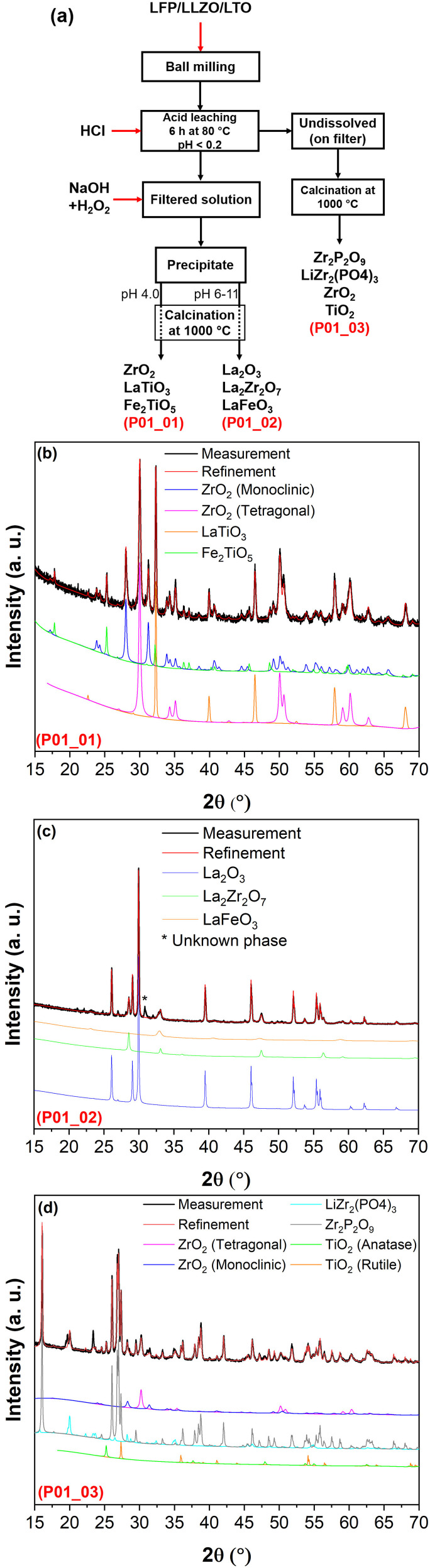
(a) Flow chart of the acid leaching/alkali precipitation process in HCl (for leaching) with pH<0.2; XRD patterns of recovered products (b) after alkali precipitation at pH 4.0 (P01_01), (c) after alkali precipitation at pH 6–11 (P01_02), (d) on filter which were undissolved during acid leaching (P01_03). All recovered products were calcinated at 1000 °C for 12 h before XRD measurement. For a better presentation, an ID has been assigned to each pattern, which can be seen in left down of each pattern by red letter.

**Figure 2:**
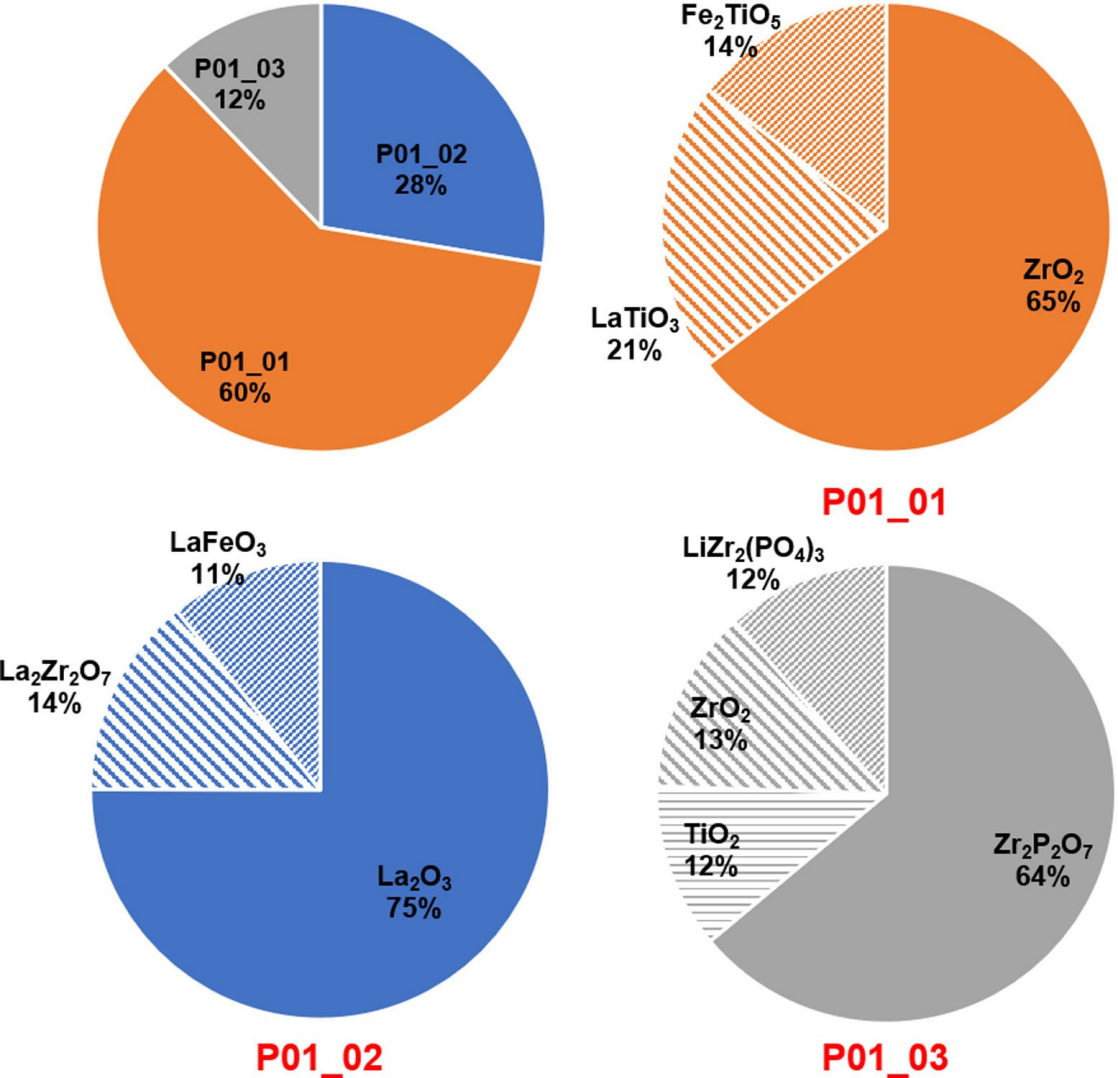
(a) Relative weight fraction of the products and each phase within different products after acid leaching/alkali precipitation process at HCl (for leaching) with pH<0.2. All recovered products were calcined at 1000 °C for 12 h

In the first leaching/precipitation process, the LFP/LLZO/LTO mixture has been subjected to a selective leaching in HCl solution with pH<0.2. Figure 1a provides an overview about the leaching/precipitation processes and the obtained products. It should be taken into consideration that all of the mentioned products were obtained after calcinating the precipitates. These observations reveal that ≈85 wt % of the powder mixture dissolved in the acidic solution, while a small fraction was left undissolved in form of a black powder (mainly consisting of Zr, phosphate and Ti ions), which later was collected by a filter (see Figure 2). To recover the dissolved ions within the filtered solution, an adequate amount of NaOH was added to increase the pH level. Simultaneously, a small quantity of H_2_O_2_ was used to oxidize Fe^2+^ to Fe^3+^ and assist to form the precipitate. The final products after the acid leaching/alkali precipitation are shown in Figures 1b–d and also summarized in Table 1 (for the detailed weights of each product, see Table S1).

**Table 1:** A summary of obtained phases at different steps of acid leaching/alkali precipitation using concentrated HCl (pH<0.2) for leaching. The data has been extracted from the Rietveld refinement of the respective XRD patterns.

	Phase	Space group	Lattice parameters [Å]	wt %
After alkali precipitation at pH 4.0 **(P01**_**01)**	ZrO_2__Tetragonal	*P*4_2_/*nmc*	*a*=3.581(1), *c*=5.181(1)	38
	ZrO_2__Monoclinic	*P*12_1_/*c*1	*a*=5.126(1), *b*=5.173(1), *c*=5.309(1), *β*=98.99(1)°	26
	LaTiO_3_	*Pm*‐3 *m*	*a*=3.879(1)	21
	Fe_2_TiO_5_	*Bbmm*	*a*=9.789(1), *b*=9.984(1), *c*=3.722(1)	14
After alkali precipitation at pH 6–11 **(P01**_**02)**	La_2_O_3_	*P*‐3 *m*1	*a*=3.936(1), *c*=6.133(1)	75
	La_2_Zr_2_O_7_	*Fd*‐3 *m*	*a*=10.815(1)	14
	LaFeO_3_	*Pm*‐3 *m*	*a*=3.843(1)	11
Undissolved during acid leaching (after calcination) **(P01**_**03)**	Zr_2_P_2_O_9_	*I*12/*m*1	*a*=10.257(1), *b*=6.592(1), *c*=10.025(1), *β*=95.39(1)°	64
	LiZr_2_(PO_4_)_3_	*P*12_1_/*n*1	*a*=8.886(1), *b*=8.962(1), *c*=12.405(2), *β*=89.67(2)°	12
	ZrO_2__Tetragonal	*P*4_2_/*nmc*	*a*=3.586(1), *c*=5.205(1)	7
	ZrO_2__Monoclinic	*P*12_1_/*c*1	*a*=5.101(2), *b*=5.206(3), *c*=5.336(3), *β*=98.67(3)°	6
	TiO_2__Rutile	*P*4_2_/*mnm*	*a*=4.602(1), *c*=2.971(3)	7
	TiO_2__Anatase	*I*41/*amd*	*a*=3.790(1), *c*=9.544(2)	5

By adding NaOH (+H_2_O_2_) to the filtered solution (previously leached at pH<0.2), the first precipitate formed at a pH level of 4.0. According to Table 1 and Figure 2b, the obtained recyclate (after calcination at 1000 °C) at this step contains ≈65 wt % of ZrO_2_.

The obtained zirconium oxide is a mixture of monoclinic and tetragonal modifications of ZrO_2_ (Table 1). The other 35 wt % belong to perovskite‐type La_2/3_TiO_3_ and Fe_2_TiO_5_ (Figure 2). In fact, at this step, almost half of the total Zr has been extracted in form of ZrO_2_ (recovered Zr moles per 1.0 g of the initial mixture: 8.89×10^−4^ mol; total available Zr moles in 1.0 g of the initial mixture: 1.88×10^−3^). However, the co‐existence of Ti and Fe in the recovered ZrO_2_ is not desirable for the purpose of recycling of LLZO. Nevertheless, it is worth noting that most of the extractable Ti was also extracted in this step (the amount of Ti‐related phase, which were obtained in further steps, was marginal as compared to what was obtained here).

In the next step, the second product forms at the pH level of around 6 up to pH 11. The product phase at this step contains mainly La_2_O_3_ (≈75 wt %), while the pyrochlore phase of La_2_Zr_2_O_7_ has also been detected with a weight percent of ≈14 %. This phase is not problematic during the recycling of LLZO, since it can easily be converted to LLZO at temperatures higher than 1000 °C.^[20]^ However, together with La_2_O_3_ and La_2_Zr_2_O_7_, some amount of Fe in form of LaFeO_3_ can be observed (Figures 1c and 2 and Table 1) which can be a source of impurity (due to Fe) when it comes to recycling of LLZO.

By leaching the mixture using such highly concentrated HCl, only a small amount of the mixture sample was left undissolved (≈0.07 g per 1.0 g of the mixture (Table S1 and Figure 2)). The XRD measurements suggest that the undissolved powders contain mostly Zr and phosphate, since mainly Zr_2_P_2_O_9_ (Zr_2_O(PO_4_)_2_) and LiZr_2_(PO_4_)_3_ could be detected after calcination (see Figures 1d and 2 and Table 1). Only a small fraction of Ti could be extracted in form of TiO_2_ (rutile and anatase) during this step. The results obtained here reveal that leaching the LFP/LLZO/LTO mixture in a solution with pH<0.2 does not serve to recover the desired precursor materials such as ZrO_2_ or La_2_O_3_ with acceptable purity (due to co‐precipitation of Fe and Ti along La and Zr). For this reason, it was not used further in the recycling of LLZO.

### Leaching with HCl Solution pH=1.0

**Figure 3:**
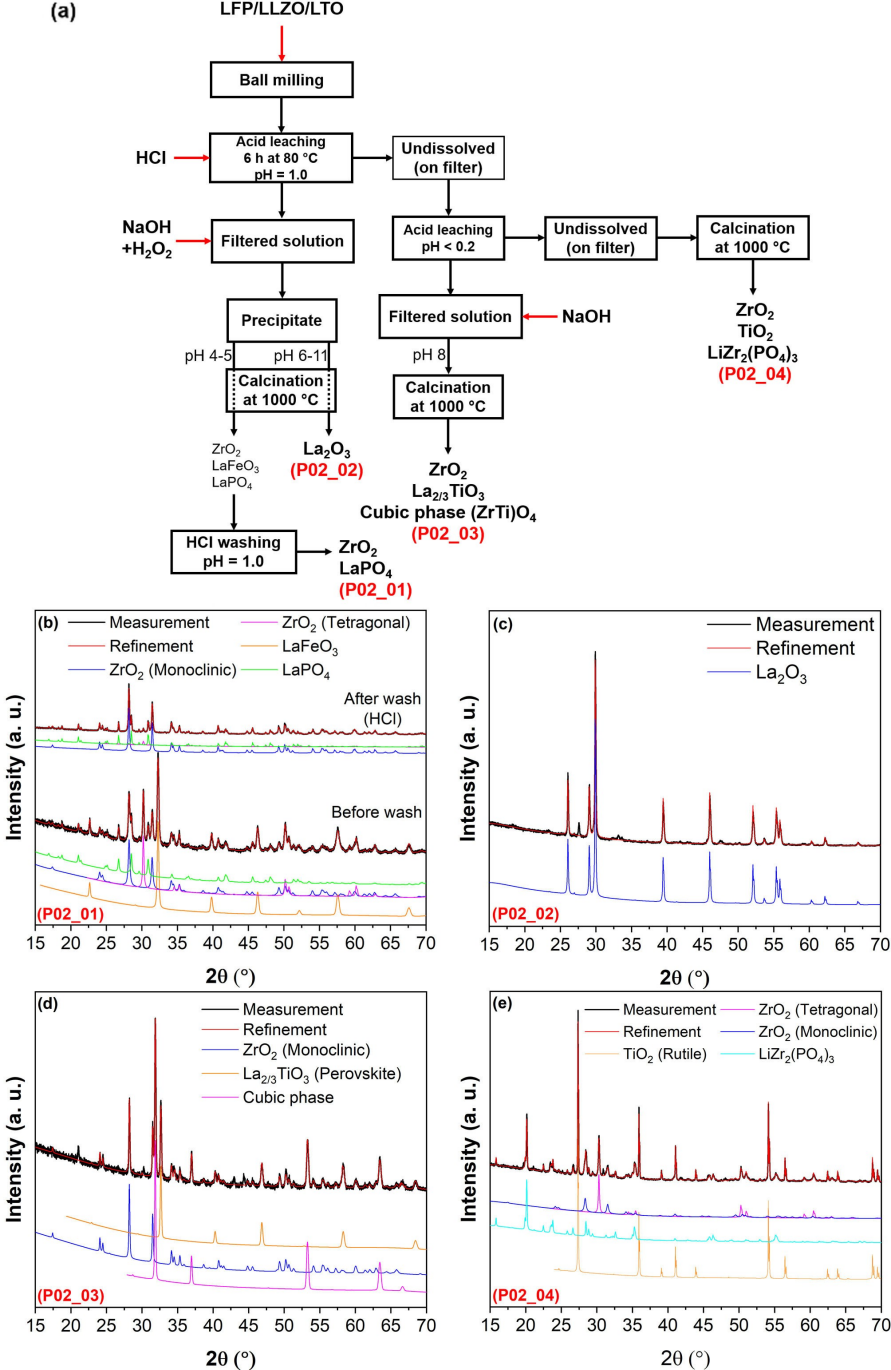
(a) Flow chart of the acid leaching/alkali precipitation process using HCl (for leaching) with pH**=**1.0; XRD patterns of recovered products (b) after alkali precipitation at pH 4–5 (P02_01), (c) after alkali precipitation at pH 6–11 (P02_02), (d) after second leaching at a pH<0.2 and precipitation at pH up to 8.0 (P02_03), (e) on filter, which were undissolved during the acid leaching processes (P02_04). All recovered products were calcinated at 1000 °C for 12 h before XRD measurement. For a better presentation, an ID has been assigned to each pattern, which can be seen in left down of each pattern by red letter.

Here, the mixture of LFP/LLZO/LTO has been first leached in a HCl solution with a pH of 1.0 (less concentrated than the previous leaching process with pH<0.2). Then, the undissolved powders were leached once more using a more concentrated HCl solution (pH<0.2, see Figure 3a).

**Figure 4:**
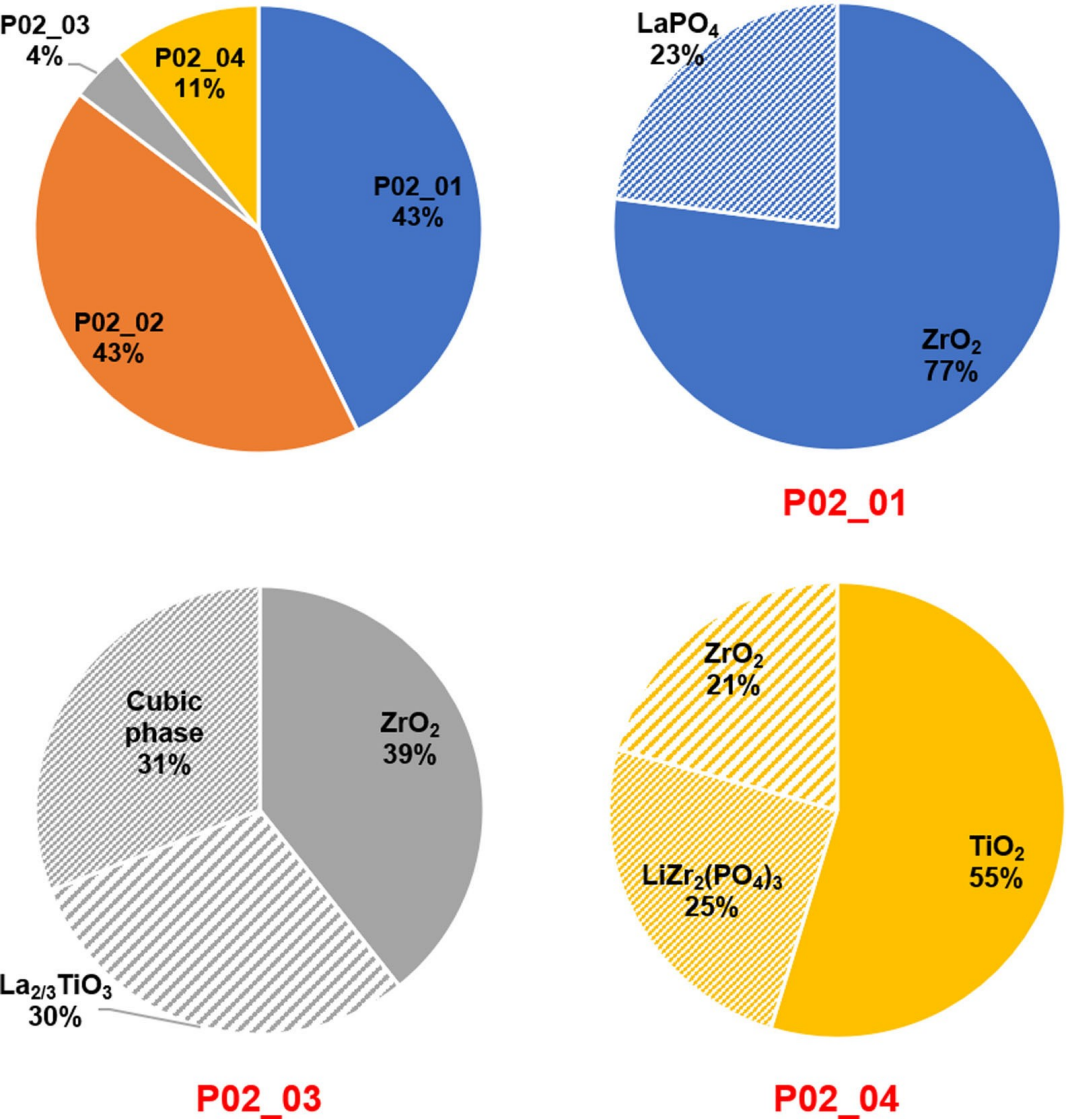
Relative weight fraction of the products and each phase within different products after acid leaching/alkali precipitation process at HCl (for leaching) with pH**=**1.0. All recovered products were calcined at 1000 °C for 12 h.

In this leaching/precipitation process, 86 wt % of the obtained products were formed in the first leaching/precipitation process (P02_01 and P02_02 in Figure 3a), according to Table S1 and Figure 4. Therefore, in the first leaching step in the solution with pH 1.0, most of Zr and all of La were dissolved in the solution. Over the alkali precipitation Zr, P, Fe and only a small fraction of La tend to precipitate at lower pH values of around 4–5, while at higher pH levels, mainly La precipitates (see Figures 3b,c and 4 and Table 2). In fact, during the alkali precipitation at pH values of 4–5, a combination of 56 wt % ZrO_2_ (with tetragonal and monoclinic structures), 25 wt % perovskite LaFeO_3_ and 18 wt % LaPO_4_ phases formed after calcination (P02_01 phase in Figure 3, relative weight percentages can be found in Table 2).

**Table 2:** A summary of obtained phases at different steps of acid leaching/alkali precipitation using HCl with pH**=**1.0) for leaching. The data has been extracted from the Rietveld refinement of the XRD patterns.

	Phase	Space group	Lattice parameters [Å]	wt %
After first alkali precipitation at pH 4.0 **(P02**_**01)**	ZrO_2__Tetragonal	*P*4_2_/*nmc*	*a*=3.596(1), *c*=5.189(1)	40
	ZrO_2__Monoclinic	*P*12_1_/*c*1	*a*=5.150(1), *b*=5.200(1), *c*=5.316(1), *β*=99.106(4)°	16
	LaFeO_3_	*Pm*‐3 *m*	*a*=3.918(1)	25
	La_2_PO_4_	*P12_1_/n1*	*a*=6.847(1), *b*=7.080(1), *c*=6.517(1) *β*=103.323(6)°	18
After first alkali precipitation at pH 6–11 **(P02**_**02)**	La_2_O_3_	*P*‐3 *m*1	*a*=3.936(1), *c*=6.133(1)	75
After second alkali precipitation at pH 8.0 **(P02**_**03)**	ZrO_2__Monoclinic	*P12_1_/c1*	*a=5.144(1), b=5.191(1), c=5.316(1), β=99.215(4)°*	39
	La_2/3_TiO_3_	*Pm‐3 m*	*a*=3.876	30
	ZrTiO_4__Tetragonal	*P*4_2_/*nmc*	*a*=3.586(1), *c*=5.205(1)	7
Undissolved during acid leaching (after calcination) **(P01**_**03)**	ZrO_2__Monoclinic	*P*12_1_/*c*1	*a*=5.101(2), *b*=5.206(3), *c*=5.336(3), *β*=98.67(3)°	6
	TiO_2__Rutile	*P*4_2_/*mnm*	*a*=4.602(1), *c*=2.971(3)	7

It is important to note that the perovskite phase of LaFeO_3_ can easily be washed out by a relatively low concentrated hydrochloric acid such as 0.01 m HCl (as can be seen in Figure 3b). Thus, we can obtain ZrO_2_ with a purity close to 80 wt % (Figure 4). Further washing of the ZrO_2_ product even in more concentrated acids (e. g., 5 m H_2_SO_4_ at 80 °C) did not serve to remove LaPO_4_. EDX measurements (in Table 3) confirmed that no remarkable amount of Fe could be detected after washing the powders (P02_01). However, some P and La have been found through the EDX measurement, which is due to existence of the LaPO_4_ phase. Note that the average atomic percentages of La (≈17 at.%, see Table 3) is less than that of P (≈25 at.%, see Table 3). This may suggest that a small amount of phosphate is present as an amorphous phase which cannot be detected by XRD, while the rest formed the LaPO_4_ phase.

**Table 3:** The results of EDX analysis on the product after first leaching in a HCl solution with pH**=**2.0 and precipitated at pH pH 6–11 (P03_02). The products were calcinated at 1000 °C prior to measurements.

Alkali precipitation pH range		Al−K	P−K	Ti−K	Fe−L	Zr−L	La−L
4–5 **(P02**_**01)** after HCL wash	Average wt %	0.288	10.13	0.186	0	64.127	25.256
	wt % error	±0.02	±0.186	±0.132	0	±1.221	±0.397
	Average atomic %	1.304	25.665	0.327	0	55.268	17.434
	Atomic% error	±0.07	±0.473	±0.233		±1.096	±0.247
6–11 **(P02**_**02)**	Average wt %	2.433	0.071	0.402	0.239	0.076	96.779
	wt % error	±0.068	±0.071	±0.148	±0.053	±0.286	±0.658
	Average atomic %	11.198	0.278	1.043	0.522	0.10.3	86.856
	Atomic% error	±0.313	±0.289	±0.383	±0.118	±0.392	±0.59

The next fraction of the precipitates was observed after increasing the pH of the solution from 6 up to 11 (Figure 3a). XRD measurements (Figure 3) suggest that high‐purity La_2_O_3_ has been formed at this step (after calcination). EDX results in Table 3 also confirm that this product mainly consists of La with marginal Fe, Ti, Zr and P impurities. However, some noticeable Al can also be found (Table 3) which is due to the fact that Al has previously been used as a dopant (as a cubic structure stabilizer) for preparing the initial cubic LLZO.^[21]^ Co‐precipitation of Al and La would be of interest, since this Al can be incorporated into the LLZO structure during the sintering process of making recycled cubic LLZO.

The rest of the powders which were not dissolved in the leaching solution (pH=1.0) was subjected to the second leaching step (Figure 3a) in a concentrated HCl solution with a pH lower than 0.2. It is worth noting that most of Zr and La had already been extracted during the previous step. Therefore, only ≈15 wt % of the final products (see Figure 4 and Table S1) originate from the second leaching step (P02_03 and P02_04 in Figure 3). Most of the powders could not be dissolved over the second leaching and were collected on the filter paper. Then, they formed mainly TiO_2_ (≈55 wt %), but also LiZr_2_(PO_4_)_3_ (≈25 wt %) and ZrO_2_ (≈21 wt %) (P02_04 in Figures 3e and 4) once the undissolved (in the second leaching step) powders were calcined. For recovery of the dissolved particles (in the second leaching at pH<0.2), the pH of the acidic solution was increased up to 8.0 (no precipitate formed over increasing the pH higher than 8.0) and then the precipitates were calcined at 1000 °C (note that only ≈4 wt % of the final products are the result of this process; see Figure 4). The XRD measurements and Rietveld refinements show that the product of this step consists of ≈39 wt % monoclinic ZrO_2_, 30 wt % La_2/3_TiO_3_ and the remaining 31 wt % are a cubic phase with a space group *Fm*‐3 *m* and a lattice parameter of *a*=4.861(1) Å. The closest match to this phase was found to be cubic ZrO_2_. However, cubic ZrO_2_ can normally be observed below its melting point (2800 K) up to 2640 K.^[22]^ Moreover, the lattice parameter of the obtained cubic phase strongly deviates from pure cubic ZrO_2_ found in the literature (4.861(1) Å (Table 2) versus 5.145(8) Å found in Ref. [23]). The structure of this cubic phase seems to be more similar to the fluorite‐type structure rather than rock salt. Therefore, we hypothesize that Ti was incorporated into ZrO_2_ and stabilized the structure to make a fluorite‐type (ZrTi)O_4_ phase. This could explain the reduction of the lattice parameter of this cubic phase compared to cubic ZrO_2_.^[24]^

### Leaching with HCl Solution pH=2.0

**Figure 5:**
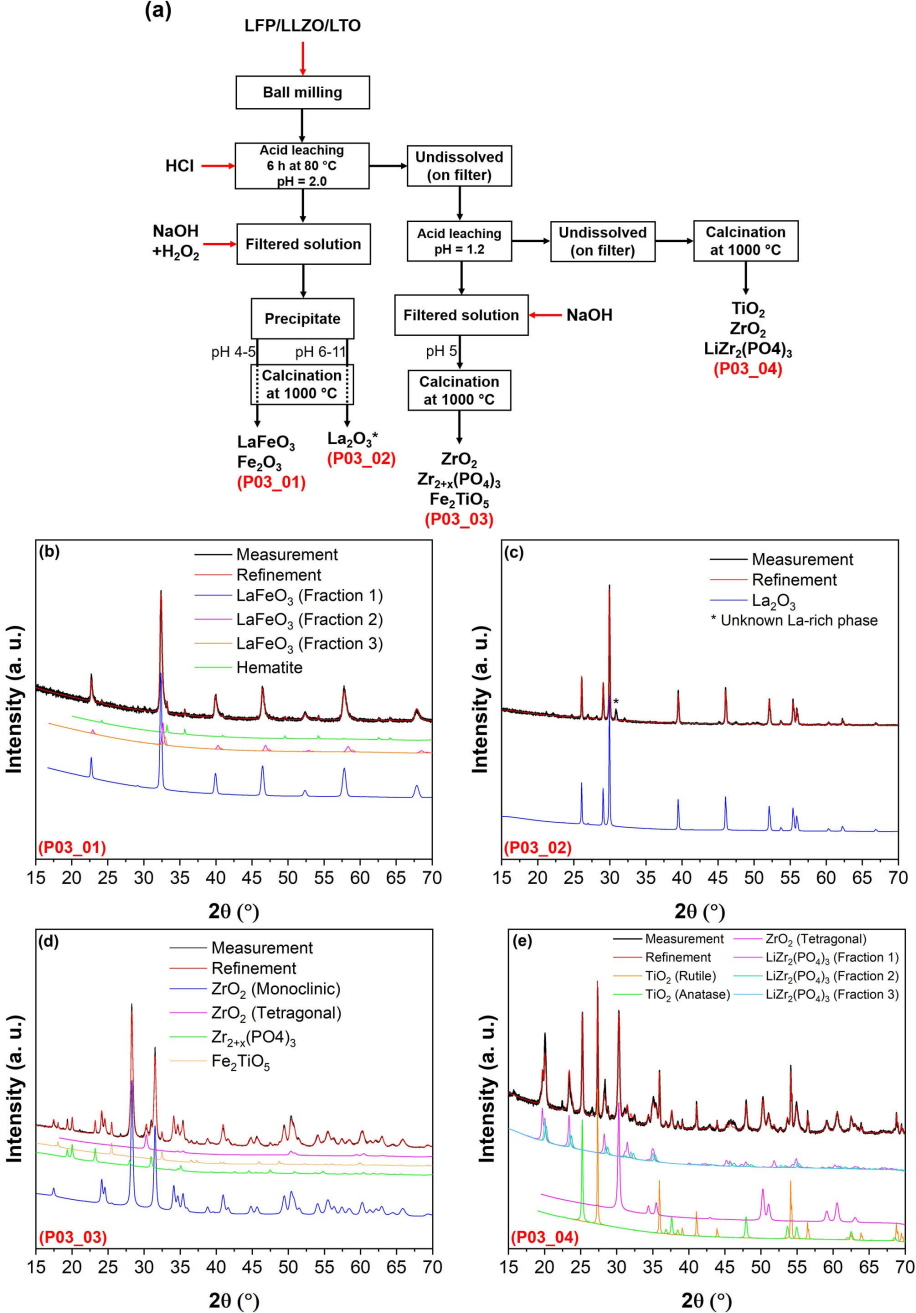
(a) Flow chart of the acid leaching/alkali precipitation process using HCl with pH=2.0 for leaching; XRD patterns of recovered products (b) after alkali precipitation at pH 4–5 (P03_01), (c) after alkali precipitation at pH 6–11 (P03_02), (d) after second leaching at a pH=1.2 and precipitation at pH up to 5.0 (P03_03), (e) on filter which were undissolved during the acid leaching processes (P03_04); (f) relative weight fraction of each phase. All recovered products were calcined at 1000 °C for 12 h before XRD measurement. For a better presentation, an ID has been assigned to each pattern, which can be seen in left down of each pattern by red letter.

**Figure 6:**
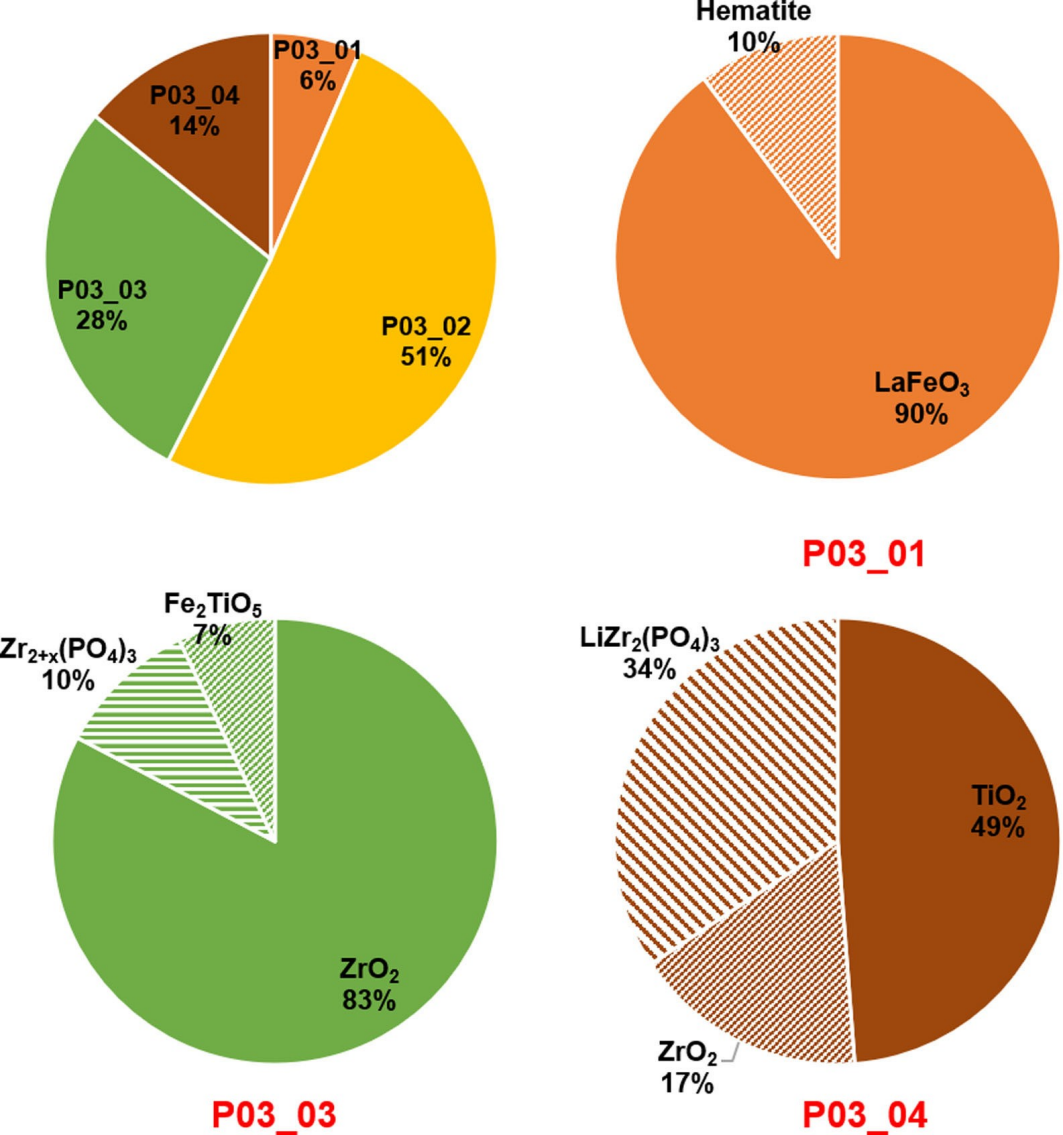
Relative weight fraction of the products and each phase within different products after acid leaching/alkali precipitation process at HCl (for leaching) with pH**=**2.0. All recovered products were calcined at 1000 °C for 12 h.

Leaching the mixture of LFP/LLZO/LTO in a low‐concentration HCl solution with a pH of 2.0 results in dissolving mainly La and Fe. In fact, by alkali precipitation at pH levels in the range of 4–5 (after leaching at pH 2.0), co‐precipitation of Fe and La is indicated from the formation of perovskite LaFeO_3_ after calcination (Figure 5b, f and Table 4). Furthermore, a small amount of Fe_2_O_3_ (≈10 wt %) formed together with LaFeO_3_ (Figures 5b, 5 f and Table 4). The majority of the LaFeO_3_ phase has a lattice parameter of 3.903(1) Å; however, two minor fractions of LaFeO_3_ could also be detected in the XRD patterns with different lattice parameters of 3.871(1) Å and 3.835(1) Å, respectively (see Table 4). Note that the LaFeO_3_/Fe_2_O_3_ product (P03_01) amounts to only 6 wt % of the total obtained products, while ≈51 wt % belong to La_2_O_3_ (P03_02 in Figure 6, Table S1). Upon increasing the pH from 6 to 11, mainly La would precipitate, whose oxide La_2_O_3_ can be detected by XRD after calcinating the precipitates at this pH level. The purity of the recovered La_2_O_3_ seems to be very high according to the Rietveld analysis of the XRD data shown in Figure 5c. Only one low intensity reflection (marked by * in Figure 5c) could be detected which is not related to La_2_O_3_.

**Table 4:** A summary of obtained phases at different steps of acid leaching/alkali precipitation using HCl solution with pH**=**2.0 for leaching. The data has been extracted from the Rietveld refinement of the XRD patterns.

	Phase	Space group	Lattice parameters [Å]	wt %
After first alkali precipitation at pH 4–5 **(P03**_**01)**	LaFeO_3_ (3 fractions)	*Pm*‐3 *m*	*a=3.903(1) (Fraction 1) a=3.871(1) (Fraction 2) a=3.835(1) (Fraction 3)*	90
	Fe_2_O_3_ (Hematite)	*R‐3cH*	*a=5.022 (1), c=13.703 (1)*	10
After first alkali precipitation at pH 6–11 **(P03**_**02**	La_2_O_3_	*P‐3 m1*	*a=3.937(1), c=6.133(1)*	100
After second alkali precipitation at pH 5.0 **(P03**_**03)**	ZrO_2__Monoclinic	*P12_1_/c1*	*a=5.134(1), b=5.175(1), c=5.320(1), β=98.990(4)°*	39
	ZrO_2_ (Tetragonal)	*P4_2_/nmc*	*a=3.608(9), c=5.109(26)*	4
	Zr_2+x_(PO_4_)_3_, x≈0.4	*R‐3cH*	*a*=8.855(1), *c*=22.845(3)	10
	Fe_2_TiO_5_	*Bbmm*	*a=5.134(1), b=5.175(1), c=5.320(1)*	7
Undissolved during acid leaching (after calcination) **(P01**_**03)**	TiO_2_ (Rutile)	*P*4_2_/*mnm*	*a*=4.603(1), *c*=2.971(3)	27
	TiO_2_ (Anatase)	*I41/amd*	*a*=3.791(1), *c*=9.551(3)	22
	ZrO_2_ (Tetragonal)	*P*42/*nmc*	*a*=3.574(1), *c*=5.208(3)	17
	LiZr_2_(PO_4_)_3_ (3 fractions)	*R*‐3*cH*	*a*=8.837(1), *c*=22.224(3) (fraction 1) *a*=8.798(2), *c*=21.347(3) (fraction 2) *a*=8.866(1), *c*=21.615(5) (fraction 3)	34

However, the EDX analysis presented in Table 5 confirms that the formed La_2_O_3_ has a very high purity (weight fraction of La ≈99 %).

**Table 5:** The results of EDX analysis on the product after first leaching in a HCl solution with pH**=**2.0 and precipitated at pH pH 6–11 (P03_02). The products were calcinated at 1000 °C prior to measurements.

	Al−K	P−K	Ti−K	Fe−L	Zr−L	La−L
Average wt %	0.081	0.086	0.481	0.214	0	99.084
wt % error	0.028	0.085	0.124	0.053	0	0.625
Average atomic %	0.406	0.379	1.365	0.516	0	97.243
Atomic% error	0.142	0.373	0.35	0.125	0	0.613

Following this strategy, all of the extractable lanthanum and most of iron could be extracted. The next step was to try to extract Zr. Therefore, the undissolved powders were leached once again in a stronger acid with a pH level of 1.2. After leaching the mixture, the filtered solution was subjected to alkali precipitation by adding sufficient amount of NaOH to increase the pH up to 5.0.

The products (P03_03) after calcination of the precipitates include mostly ZrO_2_ (about 83 wt %). In addition, a zirconium phosphate phase (≈10 wt %) and Fe_2_TiO_5_ (≈7 wt %) could also be detected by XRD (Figures 5d and 6 and Table 4). The purity of the obtained ZrO_2_ (mainly monoclinic) is considerable (≈83 wt %). Nevertheless, the whole products at this stage was determined to be only 28 wt % of the total products that formed in the whole acid leaching/alkali precipitation process (Figure 6, Table S1). In fact, still some noticeable amount of Zr was left un‐extracted within the undissolved powder.

Finally, the undissolved powder after the second leaching step has been calcined, which has a portion of ≈14 wt % (Figure 6, Table S1) as compared to other products in the leaching/precipitation process described in Figure 5a. As expected, Ti was only partially dissolved in the previous leaching steps and therefore, almost 50 wt % of the undissolved powder (after calcination) is TiO_2_ in rutile and anatase modifications (Figures 5e and 6 and Table 4). The rest of the products at this step (P03_04) are 34 wt % of LiZr_2_(PO_4_)_3_ and 17 wt % of ZrO_2_ (tetragonal) phases, as shown in Figures 5e and 6 and Table 4.

### Efficiency of the Acid Leaching/Alkali Precipitation Processes

Here we will briefly compare the previously stated leaching experiments (at pH<0.2, pH=1.0 and pH=2.0; details can be found in Figures 1a, 3a and 5a, respectively) and determine which process has a higher efficiency towards making high‐purity La_2_O_3_ and ZrO_2_. From the results reported in the previous sections, it can be concluded that lanthanum and iron tend to dissolve even in less concentrated HCl, while zirconium, phosphate and titanium mainly dissolve in concentrated solutions (pH≤1.0). Furthermore, carbon (added for improving the electronic conductivity) does not significantly dissolve in the acidic solutions due to its hydrophobic nature.^[25]^ It is expected that PVDF has been washed out by acetone (each intermediate product was washed with acetone prior to calcination, as discussed in the Experimental Section). Likewise, the solubility of PVDF (added as a binder) in the examined solutions is expected to be negligible since HCl is not to be known for dissolving PVDF.^[26]^

Phosphate tends to dissolve in the solution at a pH level of around 1.0. In fact, through leaching the mixture in a solution with a pH=1.0, most of the phosphate phase can be dissolved in the solution and form LaPO_4_ after calcination (Figure 3). However, considering the products from leaching the LFP/LLZO/LTO (milled) powder mixture at concentrated HCl solution (pH<0.2), it can be concluded that the phosphate phase stayed mainly undissolved and was converted to Zr_2_P_2_O_7_ (Zr_2_O(PO_4_)_2_) and LiZr_2_(PO4)_3_ during calcination, while no other phosphate‐related phase(s) could be detected after the precipitation step of the filtered solution (see Figure 1). On the other hand, the concentration of HCl at pH=2.0 seems not to be high enough to leach phosphate (Figure 3). This was unexpected, since previous studies reported that an HCl solution at pH 2.0 could dissolve LFP and that the dissolved phosphate could be recovered as FePO_4_.^[27]^ This may arise due to altered solubilities in complex systems. In this respect, the solubility of [PO_4_]^3−^ is further influenced by the availability of other ions, for example La^3+^, which can form additional precipitates such as LaPO_4_. This shows that recycling processes should be considered in the context of the real complex system (e. g., LFP/LLZO/LTO), in which different elements may affect each other's solubility through co‐precipitation. Nevertheless, a phosphate (Zr_2+x_(PO_4_)_3_) phase could be formed once the undissolved powders were further leached for the second time by a solution with a pH of around 1.0 (see Figure 5).

Table 6 provides a summary about the total moles of recovered elements versus their expected moles per 1.0 g of the mixture. Note that the data are referring to the recovery of the elements in any form and not within a specific composition (e. g., ZrO_2_ or La_2_O_3_). As can be seen in Table 6, the highest recovery of La and Zr of 82 wt % and 87 wt %, respectively, can be found for the leaching of the mixture in a solution with a pH 1.0. Furthermore, almost 77 wt % of La and Zr could be recovered upon leaching the mixture in a concentrated HCl solution with a pH<0.2. The least recovery of La and Zr (56 wt % and 62 wt %, respectively) was observed when the mixture was leached in a HCl with pH=2.0.

**Table 6:** Total recovery of each element at different leaching process (pH<0.2, pH**=**1.0 and pH**=**2.0), which is extracted from the phase quantification of XRD patterns. The error of the reported Recovered (%) is less than 3 % for all of the recovered elements.

		Recovered elements				
		La	Zr	P	Fe	Ti
Leaching in a concentrated HCl solution (pH<0.2)	Total recovered moles per 1.0 g of mixture	2.17E−03	1.44E−03	3.03E−04	3.69E−04	4.06E−04
	Expected moles per 1.0 g of mixture	2.82E−03	1.88E−03	4.37E−04	4.37E−04	7.58E−04
	**Recovered (%)**	**77**	**76**	**69**	**84**	**54**
Leaching in HCl solution with pH=1.0	Total recovered moles per 1.0 g of mixture	2.31E−03	1.63E−03	3.41E−04	2.91E−04	5.38E−04
	Expected moles per 1.0 g of mixture	2.82E−03	1.88E−03	4.37E−04	4.37E−04	7.58E−04
	**Recovered (%)**	**82**	**87**	**78**	**67**	**71**
Leaching in HCl solution with pH=2.0	Total recovered moles per 1.0 g of mixture	1.61E−03	1.16E−03	2.29E−04	2.33E−04	4.51E−04
	Expected moles per 1.0 g of mixture	2.85E−03	1.88E−03	4.37E−04	4.37E−04	7.58E−04
	**Recovered (%)**	**56**	**62**	**52**	**53**	**60**

We emphasize that the elemental loss here could be attributed to a combination of several reasons such as sticking of the powders to the reaction beaker, filter paper and crucible (during calcination), loss during weighing steps, and, finally, incomplete precipitation from the solution. Nevertheless, since all the three leaching/precipitation processes have been performed as similar as possible, the different amounts of loss in the different processes are assumed to be arising mainly from un‐precipitated particles (left dissolved in the solution).From the data in Table 6, it can be concluded that leaching the mixture in an HCl solution with a pH of around 1.0 would not only serve to recover the highest amount of La and Zr (as compared to other leaching conditions), but also paves the way to recover La_2_O_3_ and ZrO_2_ (Figure 3) with a significantly reduced amount of Ti and Fe impurities. In this respect, it is assumed that making recycled LLZO based on the recovered ZrO_2_ and La_2_O_3_ from the process, in which the mixture was leached in a solution with a pH=1.0, would provide LLZO with considerable purity.

### Reformation of Cubic LLZO

#### The Effects of Precursors and Leaching Process on the Purity of LLZO

In the previous sections, we have shown that it is in principle possible to extract La_2_O_3_ and ZrO_2_ from the complex mixture of LFP/LLZO/LTO. However, the purity of the recovered compounds was different at various leaching procedures. Nevertheless, we used the recovered products of La_2_O_3_ and ZrO_2_ to re‐form the LLZO phase according to the following Equation (1):

(1) $$7\;\mathrm{Li}{}_{2}\mathrm{CO}{}_{3}+3\;\mathrm{La}{}_{2}O{}_{3}+4\;\mathrm{ZrO}{}_{2}\to 2\;\mathrm{Li}{}_{7}\mathrm{La}{}_{3}\mathrm{Zr}{}_{2}O{}_{12}+7\;\mathrm{CO}{}_{2}↑{}^{\left[28\right]}$$

Therefore, P01_01, P02_01 and P03_03 (as a source for ZrO_2_) were mixed with P01_02, P02_02 and P3_02 (as a source for La_2_O_3_), respectively, to form LLZO. Since this article aims to address the recovery of the heavy elements instead of Li, which has been described in literature before,^[27b]^ the required Li_2_CO_3_ has been added from an external source. Further, extraction of such low amount of Li in form of Li_2_CO_3_ (corresponds to ≈0.26–0.52 g) as a common method[^5b^, ^27b^, ^29^] is not likely within the current experimental conditions due sufficient solubility of Li_2_CO_3_ in aqueous solutions (about 1.08 g per 100 mL and 0.69 g per 100 mL at 40 °C and 100 °C, respectively)^[30]^ as well as fairly low efficiency (less than 70 %) of the recovery process using Na_2_CO_3_ [Eq. 2].^[31]^

(2) $$2\;\mathrm{Li}{}^{+}+\mathrm{Na}{}_{2}\mathrm{CO}{}_{3}\to \mathrm{Li}{}_{2}\mathrm{CO}{}_{3}↓+2\;\mathrm{Na}{}^{+}$$

**Figure 7:**
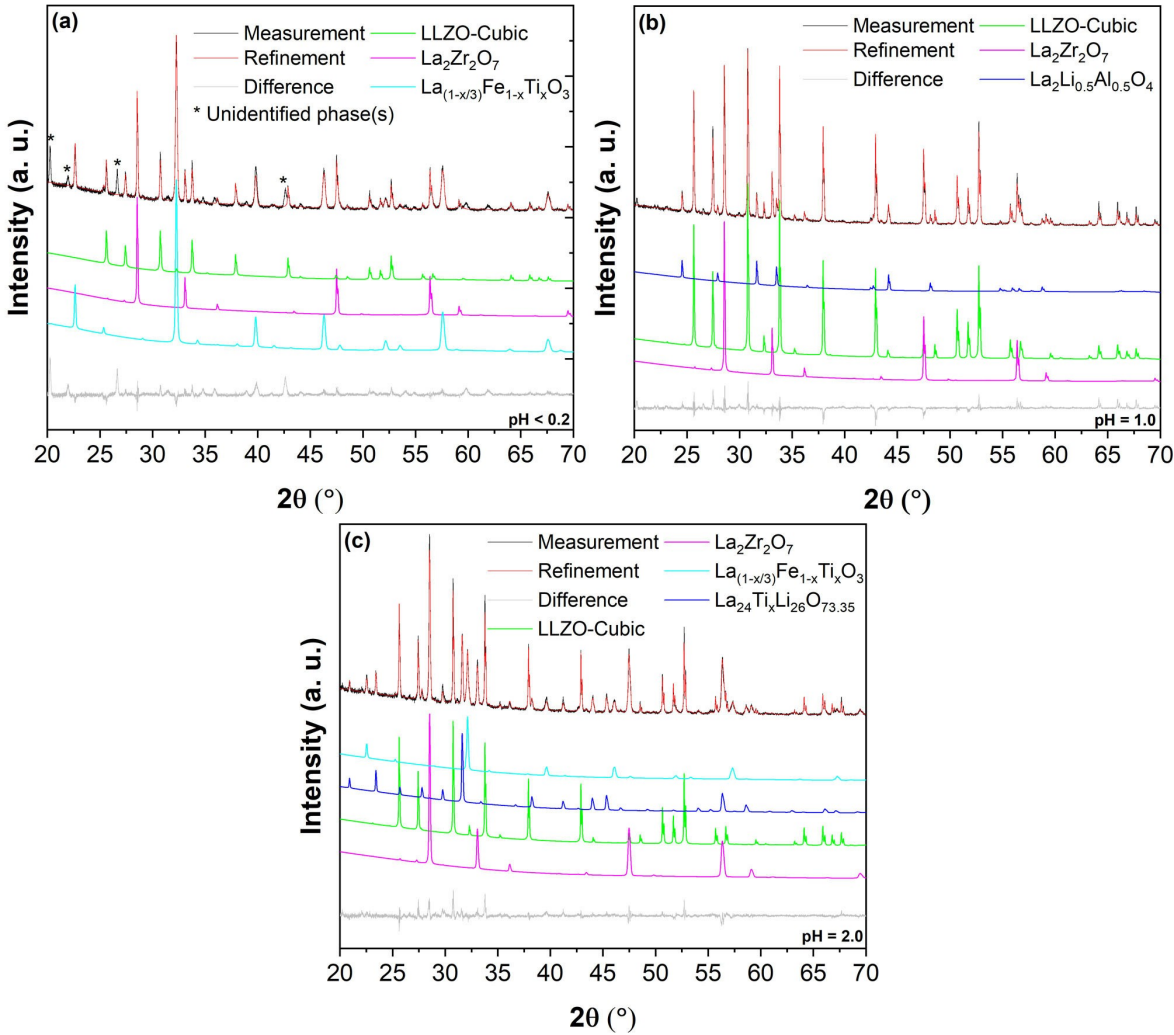
XRD patterns and Rietveld refinements of recycled LLZO using the recovered La_2_O_3_ and ZrO_2_ from different leaching processes (a) at concentrated acidic condition (pH<0.2; (P01_01) and (P01_02) were used as the precursors); (b) at pH**=**1.0, ((P02_01) and (P02_02) were used as the precursors); (c) pH**=**2.0, ((P03_02) and (P03_03) were used as the precursors).

**Figure 8:**
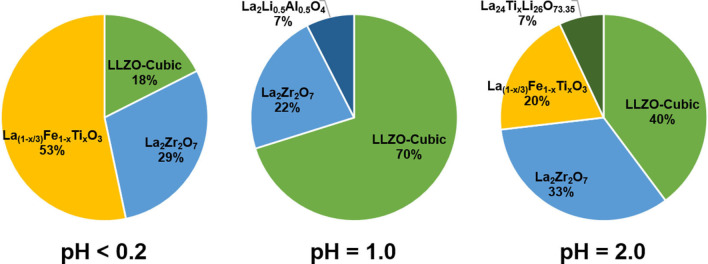
Relative weight fraction of the recycled LLZO as compared to other products at each leaching scenario (pH<0.2, pH**=**1.0, and pH**=**2.0).

We found that cubic LLZO could be formed using the recovered La_2_O_3_ and ZrO_2_ in all three cases (corresponding to three different leaching procedures). However, the purity of cubic LLZO significantly differed for different leaching scenarios, which is mainly due to the amount of Ti/Fe impurities. Figure 7a shows the XRD pattern of the recycled LLZO using the La_2_O_3_ and ZrO_2_ precursors obtained from the leaching process at pH<0.2 (P01_01 and P01_02). The results reveal that the product contains only about 18 wt % of cubic LLZO (Figure 8) and ≈29 wt % of the pyrochlore La_2_Zr_2_O_7_ phase. The formation of the pyrochlore phase is due to the instability of LLZO at high temperatures originating from loss of lithium due to it high volatility at high sintering temperatures leading to decomposition of LLZO^[32]^ and has been previously reported in several studies.^[33]^ However, La_2_Zr_2_O_7_ can be converted to LLZO upon availability of sufficient Li sources^[21c]^ and La_2_O_3_. After all, the majority of the product compound consisted of a perovskite phase, which is believed to be La_(1−x/3)_Fe_1−x_Ti_x_O_3_ phase with×close to 0. This conclusion could be drawn according to the lattice parameters of the obtained perovskite phase, which were found to be *a*=5.5426(13) Å, *b*=5.5420(9) Å, *c*=7.8251(12) Å (*Pbnm* space group, Table S4) which are very close to the lattice parameters of pure LaFeO_3_ (*a*=5.5540(1) Å, *b*=5.57677(38) Å, *c*=7.8591(9), *Pbnm* space group).^[ 34]^ Finally, an unidentified phase was also observed (marked by * in Figure 7a) in the XRD pattern of the recycled LLZO which most likely is a Ti‐rich phase since Ti was present as an impurity in the recovered ZrO_2_ (see P01_01 pattern in Figure 1ba that was used to recycle LLZO.

The lowest amount of impurities in the recycled LLZO was detected once the La_2_O_3_ (P02_02 in Figure 3) and ZrO_2_ (P02_01 in Figure 3) precursors were recovered from leaching in an acidic solution with a pH of 1.0 (following the process described in Figure 3). This was predictable and arises from the fact that the recovered La_2_O_3_ was relatively phase‐pure (see Figure 3c) and no major Fe or Ti impurities could be detected in the recovered ZrO_2_ (see Figure 3a). In this respect, no Ti‐ or Fe‐containing phases could be found in LLZO, which was recycled using the mentioned La_2_O_3_ and ZrO_2_ (see Figure 7b). Furthermore, after the recycling process, the cubic LLZO phase is dominant (≈70 wt %, see Figure 8), while only ≈22 wt % of the product consist of La_2_Zr_2_O_7_ (see Figure 8). As previously discussed, La_2_Zr_2_O_7_ is not considered to be a challenge, since it can simply be converted to cubic LLZO. Finally, approximately 7 wt % of La_2_Li_0.5_Al_0.5_O_4_ could also be detected in the XRD pattern of the recycled LLZO. The production of this phase during synthesis of LLZO has previously been reported and is suggested to originate from the alumina crucible.^[35]^ Although no other major impurities could be detected by XRD, traces of P (≈5 wt %) have been found by the EDX measurements (Table S5). However, no phosphate‐related phase(s) could be detected in the XRD pattern (Figure 7b), probably due the amorphous nature of such phase(s). It is also worth noting that the lattice parameter of the recycled LLZO (12.9688(1) Å, Table 7) is very close to that of the original LLZO (12.9567(3) Å, Table 7), suggesting that no major ion has been incorporated into the unit cell of the recycled LLZO. Furthermore, no considerable amount of Ti or Fe impurities could be detected by EDX (Table S5).

**Table 7:** A comparison between the lattice parameter of the original LLZO and the recycled LLZO phases, which were synthesized based on the recovered La_2_O_3_ and ZrO_2_ obtained from different leaching processes. The lattice parameters were extracted from the XRD data by Rietveld refinement.

	pH of the leaching solution	Space group	a parameter [Å]
Original LLZO		*Ia‐*3*d*	12.9567(3)
Recycled LLZO	<0.2	*Ia‐*3*d*	12.9791(3)
	1	*Ia‐*3*d*	12.9688(1)
	2	*Ia‐*3*d*	12.9725(1)

Figure 7c shows the XRD pattern of the recycled LLZO using recovered La_2_O_3_ (P03_02) and ZrO_2_ (P03_03) at pH=2.0 described in Figure 5. According to the Rietveld quantitative analysis, the product consists of roughly 40 wt % cubic LLZO and 33 wt % pyrochlore La_2_Zr_2_O_7_ phase as depicted in Figure 8. The recycled product is significantly less pure compared to the LLZO that was recycled using the recovered products originating from leaching at pH 1.0. This is mainly due to existence of significant amounts of impurities of Fe and Ti within the recovered precursors (see Figure 5). This resulted in formation of perovskite phase of La_(1−x/3)_Fe_1−x_Ti_x_O_3_ (≈20 wt %, Figure 8) together with the recycled cubic garnet‐type LLZO and pyrochlore La_2_Zr_2_O_7_ phase. The lattice parameters of this La_(1−x/3)_Fe_1−x_Ti_x_O_3_ phase can be found in Table S4 (*a*=5.5811(4) Å, *b*=5.5572(5) Å, *c*=7.8545(7) Å, *Pbnm*), showing that the composition is more close to LaFeO_3_, while some B‐sites may have been occupied by Ti atoms since the lattice parameters slightly differ from LaFeO_3_ found in the literature.^[34]^ Such an occupation of the B‐sites results in a change in the lattice parameters.^[36]^ The last phase found in the XRD pattern of the recycled LLZO (precursors obtained upon leaching at pH 2.0) is a tetragonal phase with a space group of *I*4/*mmm*. From the overall scattered intensity, it is estimated that this phase has a maximum relative weight fraction of ≈7 % (Figure 8).

#### Optimizing the Purity of Cubic LLZO

**Figure 9:**
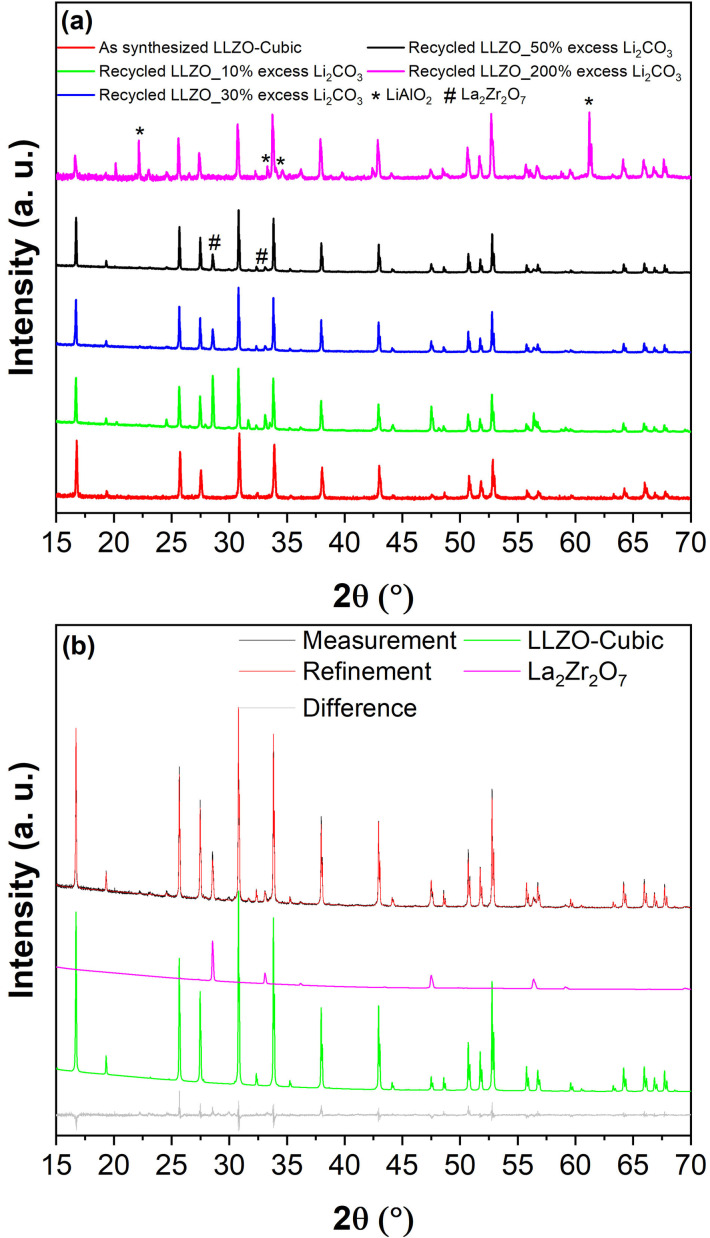
(a) XRD patterns of the recycled LLZO using different excess amounts (10 %–200 %) of Li_2_CO_3_ with La_2_O_3_ and ZrO_2_ precursors obtained from leaching at pH 1.0; (b) XRD pattern and refinement of the recycled LLZO using 50 % excess of Li_2_CO_3_ and the same precursors. For a better depiction, the calculated pattern for La_2_Li_0.5_Al_0.5_O_4_ has not been shown in part (b) due its very low overall intensity.

So far, we have shown that LLZO can be recycled; however, the best results with respect to purity of the LLZO were obtained for the case that the precursors were recovered from leaching a mixture of ball‐milled LFP/LLZO/LTO in HCl solution with a pH level of 1.0 (Figures 7 and 8). As can be seen in Figures 7b and 8, the major impurity was determined to be the pyrochlore phase of La_2_Zr_2_O_7_ (La_2+x_Zr_2−x_O_7−x/2_). The formation of this phase, which can result in a reduction of the ionic conductivity,[^11^, ^37^] can be assigned to Li loss during the sintering step.^[28]^ Nevertheless, the pyrochlore phase may be converted into LLZO by providing a sufficient excess amount of Li and La_2_O_3_ sources and high enough sintering temperatures.^[28]^ This is the strategy that we followed in order to further improve the purity of the recycled LLZO (precursors were recovered from leaching process at pH=1.0). Figure 9a provides the XRD patterns of the recycled LLZO with respect to the excess amount of Li_2_CO_3_ content (a sufficient amount of La_2_O_3_ was added according to the stoichiometry of LLZO in each case), showing that increasing the amount of excess Li_2_CO_3_ results in reduction of the relative amount of the La_2_Zr_2_O_7_ phase. However, adding too big an excess of Li_2_CO_3_ (e. g., 200 %) leads to a reaction of Li with the crucible and formation of LiAlO_2_ phase (Figure 9a). The highest purity of the cubic LLZO phase (≈90 wt %) was obtained when a 50 wt % excess Li_2_CO_3_ was used to recycle LLZO (Figure 9b and Table 8). By only adding 30 wt % excess, the purity is ≈87 wt % (Table 8), which is only slightly lower. For both samples, the impurity phase was mainly pyrochlore La_2_Zr_2_O_7_ (≈11 wt % and ≈8 wt % for 30 wt % and 50 wt % excess Li_2_CO_3_, respectively). A marginal ≈2 wt % La_2_Li_0.5_Al_0.5_O_4_ could also be detected in each case (see Table 8). Nevertheless, such a mixture of LLZO (about 90 wt % purity) is known to be able to provide sufficiently high ionic conductivity (≈0.3 mS cm^−1^).^[38]^

**Table 8:** Relative weight fraction of each phase within the recycled LLZO samples in respect to different excess amount of Li_2_CO_3._ The La_2_O_3_ and ZrO_2_ precursors were obtained from leaching a mixture of LFP/LLZO/LTO in HCl solution with pH**=**1.0 (according to Figure 3). The data is obtained from Rietveld refinement of the XRD patterns of the respective recycled LLZO samples.

Excess Li_2_CO_3_	Obtained phases [wt %]		
	LLZO‐cubic	Pyrochlore‐type phase	La_2_Li_0.5_Al_0.5_O_4_
10	70	22	7
30	87	11	2
50	90	8	2

## Conclusion

In this study, we investigated the recycling of LLZO by hydrometallurgical approaches in the context of a complex cell system of LFP/LLZO/LTO. The main focus of the study lies on the recovering of La_2_O_3_ and ZrO_2_ as pure as possible and, by that, reproducing cubic garnet‐type LLZO solid electrolyte from the recovered materials. The results reveal that LLZO and LFP are not stable even in weakly concentrated HCl solution (pH=2.0), while LTO is quite stable in more concentrated HCl solutions (pH=1.0) and can be (partially) dissolved only in very concentrated HCl solutions (pH<0.2). Furthermore, in such a complex system, co‐precipitation of the compounds, which contain different elements, is a challenge to overcome in order to recover desired compositions with high purity. For instance, iron tends to co‐precipitate with lanthanum upon leaching in weakly concentrated HCl solutions, which results in formation of perovskite LaFeO_3_ after calcination. The other example is co‐precipitation of zirconium, lanthanum, iron and phosphate at the same time when leaching the mixture at pH=1.0, leading to formation of ZrO_2_ with impurities of LaFeO_3_ and LaPO_4_. It is worth noting that the LaFeO_3_ phase can easily be removed by a further acid‐washing step. Therefore, these steps may have to be repeated further to improve the element separation. However, separation of these phases is not always that simple and usually requires further treatments. Nevertheless, upon leaching the mixture at pH≥1.0, high‐purity La_2_O_3_ could be obtained. Furthermore, using an HCl solution with a pH level of 1.0 seems to be the most suitable condition to obtain La_2_O_3_ and ZrO_2_ with a purity high enough to recover a high purity re cubic LLZO phase.

In summary, the hydrometallurgical method is a strong and cost‐efficient technique to recycle LLZO even in a complex system. It should be taken into great consideration, that one important target of ASS‐LIBs is to use Li‐metal as the negative electrode and by that increasing the potential window. Therefore, it is less likely to have such a complex oxide system (LFP/LLZO/LTO) in practical cases. However, the aim of the present study was to improve our understanding of the system from a recycling point of view in order to adapt the findings to other ASS‐LIB systems. This might be of special importance for all‐solid‐state batteries, when it comes to cradle‐to‐cradle design through using the battery waste as the feed for production of new batteries.

## Experimental Section

### Preparation of the Starting Materials

*LLZO Electrolyte*: Cubic LLZO solid electrolyte has been synthesized by solid‐state reactions adopted from the literature.[^28^, ^39^] To stabilize the cubic structure of LLZO, Al has been incorporated into LLZO to end up with a composition of Li_6.25_Al_0.25_La_3_Zr_2_O_12_.^[21b]^ Therefore, stoichiometric amounts of powder Li_2_CO_3_ (Alfa Aesar 99 %), La_2_O_3_ (Alfa Aesar 99.9 %), ZrO_2_ (Alfa Aesar 99 %) and Al_2_O_3_ (Alfa Aesar 99.9 %) were mixed and ball milled in presence of isopropanol for 18 h at a rotational speed of 250 RPM. Note that Li_2_CO_3_ has been used 20 % in excess. Moreover, La_2_O_3_ and Al_2_O_3_ were dried at 800 °C for 12 h and then weighted inside an Ar‐filled glovebox (99.999 %) prior to mixing. In the next step, the mixture has been calcined at 900 °C for 12 h under air followed by an intermediate milling under Ar at 500 rpm for 3 h without any intermediate breaking. Finally, the powders were sintered at 1100 °C for 10 h.

*LFP Cathode Material*: Carbon‐coated LFP (LiFePO_4_/C) was synthesized by solid‐state reactions according to the literature.^[40]^ Stoichiometric ratios of Li_2_CO_3_ (Alfa Aesar 99 %), iron(III) citrate hydrate (FeC_6_H_5_O_7_.xH_2_O, Alfa Aesar Fe^III^ 16.5–20 %; Fe^II^ max 5 %) and ammonium dihydrogen phosphate (NH_4_H_2_PO_4_, Alfa Aesar 98 %) were mixed by ball milling (for 18 h at 150 rpm and no intermediate breaking) using acetone as a dispersant agent. The mixture was then heated at 300 °C for 3 h under air followed by heating at 750 °C for 12 h under Ar (flow rate 0.5 SLM) with intermediate hand grinding. The obtained carbon‐coated LFP was then mixed with PVDF (Sigma Aldrich, average molecular weight of 534,000), carbon black (dried at 190 °C for 48 h) and as‐synthesized LLZO to make the LFP cathode composite material. The weight ratios considered to be LFP/C: 40 %, LLZO: 40 %, PVDF: 10 % and C: 10 %. The mixture was then milled for 2 h at a rotational speed of 120 rpm under an Ar atmosphere (the milling vial was filled inside an Ar‐filled glovebox).

*LTO Anode Material*: LTO (Li_4_Ti_5_O_12_) was prepared by solid‐state synthesis based on the literature:^[41]^ Li_2_CO_3_ (Alfa Aesar 99 %) and TiO_2_ (Alfa Aesar 99.9 %) were mixed in stoichiometric ratios (Li_2_CO_3_ was used in an excess of 10 %) by ball milling (500 rpm for 12 h using isopropanol as a dispersing agent and without any intermediate breaking). The mixture was then calcined at 800 °C for 8 h under air. To prepare the anode composite material, as‐synthesized LTO was mixed with as‐synthesized LLZO, PVDF and C in a weight ratio of LTO: 40 %, LLZO: 40 %, PVDF: 10 % and C: 10 %. Finally, the mixture was milled for 2 h under Ar (the vial was filled inside an Ar‐filled glovebox) at a rotational speed of 120 rpm.

*LFP/LLZO/LTO Mixture*: To make the desired mixture, first a large pellet (d=31.90 mm, h=2.78 mm) made from three powder layers of LFP/LLZO/LTO was uniaxially pressed together using a pressure of 15 tons for 10 min followed by an isostatic pressing by 700 kN for 90 s. The total weight of the pellet was measured to be ≈10 g; however, the LLZO solid electrolyte was determined to amount to almost 80 % of the total mass of the sample (the details of the weight ratios of the anode, cathode, electrolyte, PVDF binder and conductive carbon are listed in Table 9). Such a larger weight ratio of LLZO was found to be typical when it comes to building up stable all‐solid‐state batteries using LLZO as the solid electrolyte.^[42]^ Next, the pellet was pulverized by ball milling at a rotational speed of 500 rpm for 12 h under Ar. The XRD pattern of the final ball milled mixture as compared to as‐synthesized LFP, LLZO and LTO compounds can be found in Figure S1. It is worth mentioning that in all of the milling operations, ZrO_2_ vials and balls have been used.

**Table 9:** The weight ratios of LFP/LLZO/LTO for making a sample pellet.

Compound	Weight [g]	wt %
LLTO	8.0078	79
LTO	0.7035	7
LFP	0.6969	7
PVDF	0.3515	3
C	0.3498	3
Total weight	10.10949

Note that, to avoid local increase in temperature during milling operations, the ball milling step were done for 10 min milling intervals followed by 20 min intermediate breaking between each milling interval unless stated otherwise.

### Leaching/Precipitation Processes

The final LFP/LLZO/LTO mixture made by ball milling of the battery pellet (as described in the previous section) was leached in hydrochloric acid at different pH levels according to Figures 1 to 5. The HCl leaching agent was selected based on a modified process described in Ref. [27b] and due its flexibility to treat the LTO and LFP.^[43]^ To make the appropriate leaching solution, HCl (37 %, supplied by Roth) was mixed with deionized water (supplied by house) to obtain the desired pH. We would like to emphasize that though HCl is a very corrosive acid, making its use for large scale processes more challenging, the leaching of very stable oxides such as LLZO and LTO calls for using such strong acids over weak but more environmentally friendly leaching agents such as organic acids, which have been used for recycling of electrode materials previously.^[44]^ Throughout the experiments, the pH values were measured by a digital pH meter (Seven2Go pH/mV S2, pH accuracy ±0.01, temperature range 0–100 °C). The pH meter was calibrated by four buffer solutions with pH levels of 4.01, 7.00, 9.21 and 10.01, before every set of experiment. All the leaching processes were done at 80 °C for 6 h. Before pouring the powder mixture into the leaching solution, the solution was kept at 80 °C for some time to ensure that the temperature was homogenous throughout the solution. The weight of the sample (mixture of LFP/LLZO/LTO) was considered to be between 1–2 grams for each leaching process; however, the ratio between volume of the solution to the weight of the sample was kept constant (200 mL solution for 1 g of the mixture). The leaching processes were done under permanent rotation of a magnetic stirring bar.

After each leaching step, the solution was filtered using VWR qualitative filter paper 303 (particle retention 5–13 μm). The leftover powder on the filter paper (undissolved) was then further leached or calcined according to the flow charts depicted in Figures 1 to 5. The filtered solutions were subjected to alkali precipitation by adding sufficient amount (to adjust the desired pH level) of 1 m to 8 m NaOH solution (supplied by Merck) at room temperature under permanent rotation of a magnetic stirring bar. Normally, 1 to 2 mL of hydrogen peroxide (H_2_O_2_ 35 % supplied by Roth) was added to the solution during alkali precipitation. The pH of the solution was increased until formation of a stable precipitate and then the solution was filtered. After filtration, the pH level of the solution was further increased by further alkali precipitation to form the next stable precipitate. This process was repeated to reach a pH level of 11. In each case, the precipitates were calcined mainly at 1000 °C for 12 h under air. This helps to reform oxides of each element (e. g. La_2_O_3_, ZrO_2_, etc.) well crystallized and free of carbonates, making the obtained products suitable for reproducing the all‐oxide starting materials. It is worth noting that, prior to each calcination, the intermediate products were washed with distilled water to remove any traces of probable NaCl (expected to be formed during HCl leaching/NaOH precipitation processes) followed by washing with acetone.

## Conflict of interest

The authors declare no conflict of interest.

1

## Supporting information

As a service to our authors and readers, this journal provides supporting information supplied by the authors. Such materials are peer reviewed and may be re‐organized for online delivery, but are not copy‐edited or typeset. Technical support issues arising from supporting information (other than missing files) should be addressed to the authors.

Supporting Information

## Data Availability

The data that support the findings of this study are available from the corresponding author upon reasonable request.
