# Supplementary material for: Towards Recycling of LLZO Solid Electrolyte Exemplarily Performed on LFP/LLZO/LTO Cells
Source: ChemistryOpen. 2022 Feb 23;11(3):e202100274. doi: 10.1002/open.202100274 (PMC8889509; doi:10.1002/open.202100274)
Supplement: Supplementary file 1 — Supporting Information [file OPEN-11-e202100274-s001.pdf]

ChemistryOpen

Supporting Information

Towards Recycling of LLZO Solid Electrolyte Exemplarily Performed on LFP/LLZO/LTO Cells**

Mohammad Ali Nowroozi,* Aamir Iqbal Waidha, Martine Jacob, Peter A. van Aken, Felicitas Predel, Wolfgang Ensinger, and Oliver Clemens

1. Experimental techniques

1.1. X-ray diffraction (XRD)

X-ray diffraction was mainly used for analyzing the structure and composition of the products mainly after calcination step. The measurements were operated using a Rigaku Smartlab diffractometer in Bragg-Brentano geometry with CuK_{α} radiation (tube voltage of 40 kV) and a HyPix-3000 detector. For all of the measurements low background single crystal silicon was used as a sample holder. Data were normally recorded in an angular range between 15° to 70° for a total measurement time up to 2 h using a step size of 0.005° and a fixed divergence slit of 0.3°.

Analyses of diffraction data were performed by using the Rietveld method as implemented in TOPAS V6. The instrumental intensity distribution was determined empirically from a sort of fundamental parameters set [1] using a reference scan of LaB_6 (NIST 660a). The microstructural parameters (crystallite size and strain broadening) were refined to adjust the peak shapes. Displacement parameters were constrained to be the same for all atoms of all phases to minimize quantification errors and to account for angular dependent intensity changes induced by absorption and surface roughness.

1.2. Energy dispersive X-ray (EDX) analysis

EDX spectroscopy was used to determine the elemental composition of some of the products. The measurements were performed using a scanning electron microscope (SEM) – Zeiss Gemini DSM 982 with Schottky field emitter. The measurement parameters considered to be as follows: accelerating voltage of 10 kV, working distance of 10 mm and measuring time of 100 s. For a flat surface powder was embedded in Epofix resin (Struers) continued by microtom-polishing with a diamond knife (Diatome) using EM UC6 ultramicrotome (Leica). The samples were coated by carbon prior to measurements. The measurements were performed in area analysis mode using 10 different areas for measuring each sample. Finally, the NSS software from ThermoFischer was used to perform the measurements and to analyze the data.

2. Results

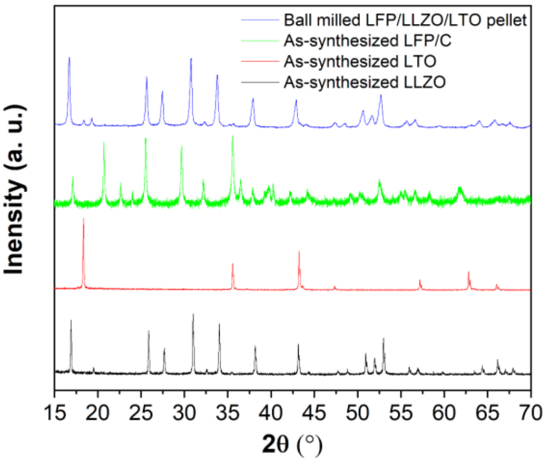

Figure S 1. XRD patterns of as-synthesized LFP, LLZO and LTO together with the pattern of milled LFP/LLZO/LTO mixture for 12h at 500 RPM. In the pattern of the milled sample, LLZO reflections are dominant due to significantly higher ratio of LLZO as compared to LFP and LTO.

Table S 1. Total obtained products at each step of acid leaching/alkali precipitation processes at different leaching conditions per 1.0 g of the initial LFP/LLZO/LTO mixture powder. The product IDs correspond to the ID numbers defined in Figure 1-3.

Product ID	pH of the leaching solution	Amount of HCl (leaching)	Amount of deionized water	Amount of NaOH (precipitation)	Amount of H_2O_2	Treatment	Total obtained weight per 1.0 g of the initial mixture after calcination (g)
P01_01				110 mL (8 M) + 150 mL DI water	1 mL	Precipitated at pH 4.0 then calcinated at 1000 °C	0.16978
P01_02	<0.2	80 mL	20 mL	11 mL (1 M)	-	Precipitated at pH 6-11 then calcinated at 1000 °C	0.36961
P01_03				-	-	Undissolved at leaching calcinated at 1000 °C	0.07520
P02_01		14 mL	200 mL	11 mL (8 M) + 5 mL (1 M)	1 mL	Precipitated at pH 4-5 then calcinated at 1000 °C	0.28583
P02_02				2.5 mL (8 M)	-	Precipitated at pH 6-11 then calcinated at 1000 °C	0.28453
P02_03	1.0	50 mL	70 mL	64 mL (8 M)	1 mL	Undissolved leached for the second time at pH<0.2 then precipitated at pH 8.0 and finally calcinated at 1000 °C	0.02621
P02_04				-	-	Undissolved at second leaching calcinated at 1000 °C	0.07225
P03_01		3	200	6 mL (8 M) + 2 mL (1 M)	1 mL	Precipitated at pH 4-5 then calcinated at 1000 °C	0.03071
P03_02				2 mL (8M)	-	Precipitated at pH 6-11 then calcinated at 1000 °C	0.24357
P03_03	2.0	7 mL	100 mL	5 mL (8 M)	1 mL	Undissolved leached for the second time at pH 1.2 then precipitated at pH 5.0 and finally calcinated at 1000 °C	0.13548
P03_04				-	-	Undissolved at second leaching calcinated at 1000	0.06732

Table S 2. The detailed results of EDX analysis on the product after first leaching in a strong acid (pH = 1.0) and precipitated at pH 4-5 (P02_01).

		Al-K	P-K	Ti-K	Fe-L	Zr-L	La-L
Area 1	wt%	0.02	11.23	0.14	0	71.1	17.51
	wt% error	±0.04	±0.21	±0.13	0	±1.25	±0.36
	atomic%	0.05	28.52	0.23	0	61.29	9.91
	atomic% error	±0.11	±0.53	±0.21	0	±1.08	±0.2
Area 2	wt%	0.06	10.28	0.1	0	71.64	17.92
	wt% error	±0.04	±0.21	±0.12	0	±1.25	±0.36
	atomic%	0.17	26.55	0.17	0	62.79	10.32
	atomic% error	±0.11	±0.53	±0.21	0	±1.1	±0.21
Area 3	wt%	0	12.25	0.18	0	70.95	16.62
	wt% error	0	±0.21	±0.13	0	±1.27	±0.36
	atomic%	0	30.5	0.29	0	59.98	9.22
	atomic% error	0	±0.53	±0.21	0	±1.07	±0.2
Area 4	wt%	0	10.7	0.19	0	72.91	16.2
	wt% error	0	±0.21	±0.13	0	±1.3	±0.37
	atomic%	0	27.3	0.31	0	63.17	9.22
	atomic% error	0	±0.53	±0.21	0	±1.08	±0.2
Area 5	wt%	0	13.5	0.21	0	68.38	17.9
	wt% error	0	±0.21	±0.13	0	±1.24	±0.36
	atomic%	0	33.06	0.34	0	56.84	9.77
	atomic% error	0	±0.52	±0.21	0	±1.03	±0.2
Area 6	wt%	0	10.36	0.16	0	73.82	15.65
	wt% error	0	±0.2	±0.12	0	±1.23	±0.35
	atomic%	0	26.55	0.27	0	64.24	8.94
	atomic% error	0	±0.52	±0.21	0	±1.07	±0.2
Area 7	wt%	0	10.5	0.36	0	71.09	18.04
	wt% error	0	±0.2	±0.13	0	±1.22	±0.36
	atomic%	0	27	0.6	0	62.06	10.34
	atomic% error	0	±0.52	±0.21	0	±1.07	±0.21
Area 8	wt%	0.01	11.11	0.28	0	69.74	18.86
	wt% error	±0.04	±0.2	±0.13	0	±1.22	±0.36
	atomic%	0.04	28.35	0.45	0	60.42	10.73
	atomic% error	±0.11	±0.52	±0.21	0	±1.06	±0.2
Area 9	wt%	0	11.37	0.03	0	71.19	17.4
	wt% error	0	±0.21	±0.13	0	±1.25	±0.36
	atomic%	0	28.82	0.06	0	61.28	9.84
	atomic% error	0	±0.53	±0.22	0	±1.08	±0.2
Area 10	wt%	2.79	0	0.21	0	0.45	96.55
	wt% error	±0.08	0	±0.17	0	±0.98	±0.73
	atomic%	12.78	0	0.55	0	0.61	86.05
	atomic% error	±0.37	0	±0.43	0	±1.32	±0.65

Table S 3. The detailed results of EDX analysis on the product after first leaching in a strong acid (pH = 1.0) and precipitated at pH 6-11 (P02_02).

		Al-K	P-K	Ti-K	Fe-L	Zr-L	La-L
Area 1	wt%	2.07	0.06	0.44	0	0	97.43
	wt% error	±0.07	±0.1	±0.15	0	0	±0.67
	atomic%	9.73	0.24	1.15	0	0	88.88
	atomic% error	±0.31	±0.43	±0.4	0	0	±0.61
Area 2	wt%	2.11	0.04	0.35	0	0	97.5
	wt% error	±0.07	±0.11	±0.16	0	0	±0.7
	atomic%	9.9	0.16	0.93	0	0	89.01
	atomic% error	±0.34	±0.46	±0.42	0	0	±0.64
Area 3	wt%	2.29	0	0.41	0	0	97.3
	wt% error	±0.06	0	±0.13	0	0	±0.59
	atomic%	10.69	0	1.09	0	0	88.22
	atomic% error	±0.27	0	±0.34	0	0	±0.53
Area 4	wt%	2.71	0.21	0.24	0.77	0	96.06
	wt% error	±0.06	±0.09	±0.13	±0.13	0	±0.58
	atomic%	12.29	0.84	0.62	1.7	0	84.55
	atomic% error	±0.27	±0.35	±0.32	±0.28	0	±0.51
Area 5	wt%	2.83	0.33	1.03	1.09	0	94.73
	wt% error	±0.05	±0.08	±0.12	±0.12	0	±0.56
	atomic%	12.51	1.28	2.56	2.32	0	81.34
	atomic% error	±0.24	±0.3	±0.29	±0.26	0	±0.48
Area 6	wt%	2.99	0.02	0.13	0.12	0.25	96.49
	wt% error	±0.08	±0.12	±0.16	±0.15	±0.99	±0.7
	atomic%	13.6	0.08	0.34	0.27	0.33	85.37
	atomic% error	±0.35	±0.49	±0.41	±0.34	±1.34	±0.62
Area 7	wt%	2.69	0.04	0.65	0	0	96.62
	wt% error	±0.08	±0.12	±0.17	0	0	±0.73
	atomic%	12.31	0.16	1.68	0	0	85.85
	atomic% error	±0.36	±0.48	±0.44	0	0	±0.65
Area 8	wt%	2.21	0.01	0.3	0.41	0	97.07
	wt% error	±0.06	±0.09	±0.13	±0.13	0	±0.61
	atomic%	10.31	0.02	0.8	0.93	0	87.94
	atomic% error	±0.28	±0.38	±0.35	±0.3	0	±0.55
Area 9	wt%	1.64	0	0.26	0	0.06	98.04
	wt% error	±0.07	0	±0.16	0	±0.89	±0.71
	atomic%	7.86	0	0.71	0	0.09	91.35
	atomic% error	±0.34	0	±0.43	0	±1.26	±0.66
Area 10	wt%	2.79	0	0.21	0	0.45	96.55
	wt% error	±0.08	0	±0.17	0	±0.98	±0.73
	atomic%	12.78	0	0.55	0	0.61	86.05
	atomic% error	±0.37	0	±0.43	0	±1.32	±0.65

Table S 4. The lattice parameters of the produced phases during recycling of LLZO using recovered La_2O_3 and ZrO_2 from different leaching processes

pH of the first leaching solution	Phases	Space group	Lattice parameters (Å)
< 0.2	LLZO_Cubic	*Ia*-3*d*	*a* = 12.9791(3)
	$La_2Zr_2O_7$	*Fd*-3*m*	*a* = 10.8073(2)
	$La_{(1-x/3)}Fe_{1-x}Ti_xO_3$	*Pbnm*	*a* = 5.5426(13), *b* = 5.5420(9), *c* = 7.8251(12)
1	LLZO_Cubic	*Ia*-3*d*	*a* = 12.9687(1)
	$La_2Zr_2O_7$	*Fd*-3*m*	*a* = 10.8092(1)
	$La_2Li_{0.5}Al_{0.5}O_4$	I4/*mmm*	*a* = 3.7754(1), *c* = 12.7606(6)
2	LLZO_Cubic	Ia-3d	*a* = 12.9725(1)
	$La_2Zr_2O_7$	*Fd*-3*m*	*a* = 10.8178(1)
	$LaFe_{1-x}Ti_xO_3$	*Pbnm*	*a* = 5.5811(4), *b* = 5.5572(5), *c* = 7.8545(7)
	$La_{24}Ti_xLi_{26}O_{73.35}$	*I*4/*mmm*	*a* = 11.9820(13), *c* = 11.9807(27)

Table S 5. The detailed results of EDX analysis on the recycled LLZO using La_2O_3 and ZrO_2 recovered from the leaching process at a pH of 1.0.

		Al-K	P-K	Ti-K	Fe-L	Zr-L	La-L
Area 1	wt%	6.65	5.87	2.04	0.18	0.52	84.74
	wt% error	±0.08	±0.13	±0.15	±	±0.87	±0.58
	atomic%	22.45	17.27	3.88	0.29	0.52	55.59
	atomic% error	±0.28	±0.39	±0.28	±0.23	±0.87	±0.38
Area 2	wt%	1.37	1.3	0.45	1.81	19.17	75.9
	wt% error	±0.04	±0.11	±0.11	±0.13	±0.85	±0.48
	atomic%	5.68	4.71	1.06	3.64	23.58	61.32
	atomic% error	±0.18	±0.41	±0.26	±0.26	±1.05	±0.39
Area 3	wt%	7.24	0.79	0.41	1.24	18.08	72.23
	wt% error	±0.07	±0.11	±0.12	±0.12	±0.8	±0.5
	atomic%	25.74	2.45	0.82	2.13	19.01	49.85
	atomic% error	±0.26	±0.33	±0.23	±0.21	±0.84	±0.34
Area 4	wt%	1.02	3.96	0.28	0	34.37	60.37
	wt% error	±0.04	±0.14	±0.12	0	±0.93	±0.46
	atomic%	3.83	13	0.6	0	38.34	44.23
	atomic% error	±0.16	±0.45	±0.24	0	±1.03	±0.33
Area 5	wt%	1.78	13.6	0.2	0.76	23.17	60.49
	wt% error	±0.05	±0.19	±0.14	±0.16	±1.15	±0.54
	atomic%	5.43	36.22	0.35	1.13	20.96	35.92
	atomic% error	±0.16	±0.52	±0.23	±0.23	±1.04	±0.32
Area 6	wt%	2.06	1.09	0.17	1.61	13.59	81.48
	wt% error	±0.05	±0.11	±0.11	±0.12	±0.83	±0.49
	atomic%	8.67	3.99	0.41	3.28	16.95	66.7
	atomic% error	±0.2	±0.39	±0.26	±0.25	±1.03	±0.4
Area 7	wt%	1.12	0.98	0.52	0.81	21.11	75.46
	wt% error	±0.04	±0.11	±0.11	±0.12	±0.84	±0.48
	atomic%	4.74	3.62	1.25	1.65	26.51	62.22
	atomic% error	±0.18	±0.41	±0.27	±0.26	±1.05	±0.4
Area 8	wt%	1.31	3.93	0.15	0.11	27.4	67.1
	wt% error	±0.04	±0.14	±0.12	±0.13	±1.06	±0.51
	atomic%	5.05	13.16	0.32	0.2	31.16	50.11
	atomic% error	±0.17	±0.48	±0.26	±0.24	±1.2	±0.38
Area 9	wt%	8.02	16.29	0.55	0.18	9.77	65.19
	wt% error	±0.09	±0.2	±0.14	±0.15	±1.17	±0.55
	atomic%	21.01	37.19	0.81	0.23	7.57	33.19
	atomic% error	±0.23	±0.46	±0.2	±0.19	±0.91	±0.28
Area 10	wt%	0.99	3.92	0.44	0.74	17.54	76.37
	wt% error	±0.04	±0.14	±0.12	±0.13	±0.97	±0.53
	atomic%	3.94	13.64	0.99	1.43	20.73	59.26
	atomic% error	±0.18	±0.48	±0.28	±0.26	±1.14	±0.41
**Average**	**wt%**	**3.156**	**5.173**	**0.521**	**0.744**	**18.472**	**71.933**
	wt% error	±0.054	±0.138	±0.124	±0.12	±0.947	±0.512

**atomic%**	**10.654**	**14.525**	**1.049**	**1.398**	**20.533**	**51.839**
atomic% error	±0.2	±0.432	±0.251	±0.213	±1.016	±0.363

3. References

[1] R. W. Cheary, A. A. Coelho, J. P. Cline, *J Res Natl Inst Stand Technol* **2004**, *109*, 1-25.
